# Changes in Sprinting and Jumping Performance During Preseason in Professional Basketball Players

**DOI:** 10.3390/jfmk10030339

**Published:** 2025-09-05

**Authors:** Álvaro de Pedro-Múñez, Tania Álvarez-Yates, Virginia Serrano-Gómez, Oscar García-García

**Affiliations:** Sport Performance, Physical Condition and Wellness Lab, Faculty of Education and Sport Sciences, University of Vigo, Campus Pontevedra, 36310 Pontevedra, Spain; alvarodpedro@gmail.com (Á.d.P.-M.); tanalvarez@uvigo.es (T.Á.-Y.); vserrano@uvigo.es (V.S.-G.)

**Keywords:** neuromuscular performance, agility, speed, preseason, basket

## Abstract

**Objectives**: Sprinting and jumping abilities are key determinants of basketball performance. This study aims to analyze changes in sprinting and jumping performance among professional basketball players during the preseason and to determine whether these adaptations are influenced by specific playing positions (Guards vs. Bigs). **Methods**: A total of 106 professional basketball players from European leagues were evaluated twice over a 6-week preseason. Neuromuscular assessments included linear sprints (5, 10, and 20 m), a change of direction test, curved sprints, and multiple jump tests: Squat Jump (SJ), Countermovement Jump (CMJ), Single-Leg CMJ (SL-CMJ) and Arm-Swing CMJ (CMJA), Single Leg Hop for Distance (SHDJ), Lateral Bound Jump (LBJ), and Single-Leg Repeated Jumps (SLRJ). The training program integrated 6–8 weekly basketball-specific technical–tactical sessions with two to three strength and conditioning sessions targeting maximal strength, power, and hypertrophy. **Results**: Players significantly improved linear and curved sprint performance, and jumping ability, particularly CMJ, CMJA, and right-leg SHDJ. Minimal changes were observed in SJ, LBJ, and SLRJ. Positional differences were small, with Guards showing greater gains in CMJA than Bigs (6.85% vs. 1.87%). **Conclusions**: A 6-week preseason training program may be associated with improvements in sprinting (linear 5, 10, 20 m, and curved sprint) and vertical jump performance (CMJ, CMJA, SHDJ) in professional basketball players, with limited influence of playing position. Guards appear to benefit more from arm-swing vertical jump development.

## 1. Introduction

In professional basketball, the ability to perform rapid sprints and changes of direction (COD) is essential for both offensive and defensive play. These high-intensity actions are fundamental to game dynamics and can significantly influence a team’s competitive performance. The preseason period provides a key critical window for enhancing these physical capacities through targeted training regimens [1].

Previous research has shown that structured preseason training can lead to significant improvements in sprinting and COD performance among basketball players. For instance, combined COD and plyometric training on sand surfaces has been associated with notable gains in sprint, jump, and agility metrics in basketball players [1]. Other studies have highlighted the importance of lower-body strength, anaerobic power, agility, sprint speed, and vertical jump performance in overall athletic effectiveness in professional basketball [2].

The physiological adaptations elicited by preseason training are multifactorial, with improvements in neuromuscular coordination, muscle strength, and power all contributing to enhancing sprinting and COD performance. A 6-week preseason training protocol targeting physiological, performance, and biochemical parameters led to significant improvements in 10 and 20 m sprint times in elite basketball players [3]. However, scientific investigations of such interventions in professional athletes remain limited, as most studies are conducted in amateur or youth populations.

Vertical jumping ability is another critical component of basketball performance, contributing to key actions such as shooting, rebounding, shot-blocking, and defensive maneuvers. Although jumping accounts for only about 1.5% of total playing time [4], these explosive actions can be decisive in determining game outcomes. Previous studies have shown that vertical jump height differentiates athletes by skill levels and playing positions and is associated with draft status and subsequent performance in professional leagues such as the NBA [5].

Despite the wide range of testing alternatives available in the scientific literature, the Squat Jump (SJ), the Countermovement Jump (CMJ), and the Arm-Swing CMJ (CMJA) are among the most widely applied assessments in basketball for evaluating lower-limb power and neuromuscular function [6,7,8]. The CMJ, which involves a rapid eccentric–concentric motion, provides valuable insights into stretch-shortening cycle efficiency and reactive strength [9]. By contrast, the SJ removes the countermovement phase, isolating concentric force production and offering a more direct measure of explosive strength [10]. These assessments are frequently applied across the competitive season due to their strong reliability for monitoring neuromuscular adaptations [11,12].

Building on these traditional vertical jump assessments and considering the multidirectional demands of basketball, it seems appropriate to also consider jumping tests across different planes, thereby capturing the players’ ability to apply force in multiple directions [13]. In line with this rationale, unilateral jump tests such as the Single-Hop for Distance Jump (SHDJ), the Lateral Bound Jump (LBJ), or the Single-Leg Repeated Jump Test (SLRJ) can also be interesting jumping tests, as single-leg tasks have been shown to more accurately represent the biomechanics of basketball sport-specific multidirectional tasks [14,15].

The SHDJ primarily evaluates horizontal force production in the sagittal plane demonstrating excellent test–retest reliability (ICC: 0.92–0.97) [16]. The LBJ, in turn, measures force application in the coronal plane with excellent intra-rater reliability (ICC > 0.96) [15], which has been associated with COD ability [17], and is able to differentiate fast from slow shufflers [18]. Finally, the SLRJ provides information on reactive strength endurance under fatigue, capturing athletes’ ability to maintain short ground contact times and effective force application across repeated jumps [19]. Together, these tests can complement vertical jump assessments by offering a multidimensional view of basketball-specific neuromuscular performance.

Nonetheless, Pehar et al. [19] proposed a similar testing battery (CMJ, Repeated Reactive Strength Ability, and Broad Jumps), while also including running vertical jumps as a measure of basketball-specific jumping ability. However, running vertical jumps have previously shown lower reliability (CV: 3–6%) compared to the consistently high reproducibility of SJ, CMJ, and CMJA (ICC: 0.93–0.97; CV: 1.4–2.6%) [6,20]. Hence, integrating both bilateral and unilateral measures into neuromuscular testing batteries appears to offer a more comprehensive assessment of the most relevant neuromuscular qualities in basketball.

Plyometric training—characterized by high-velocity movements that capitalize on the stretch-shortening cycle—has consistently been shown to improve vertical jump performance. Reported gains in CMJ height following plyometric programs range from 7.9% [21] to as much as 25% [22] with intermediate improvements such as 11.3% noted by Attene et al. [23]. Nevertheless, these interventions have largely been applied to youth and college athletes, and it remains unclear how such training impacts the neuromuscular performance of professional basketball players.

Basketball is a position-specific sport, with varying physical demands across roles. Guards generally engage in more frequent high-intensity efforts, requiring greater agility and explosive strength, whereas Bigs typically perform more strength-oriented actions such as post-ups and rebounding [24,25]. These positional differences are also reflected in jumping frequency, with Bigs averaging 49 jumps per game compared to 41 for Guards [12]. Furthermore, Bigs tend to exhibit greater braking impulse and eccentric mean force during CMJ testing, while Guards outperform them in non-concentric force when values are normalized to body mass, highlighting the importance of relative strength and power [26].

Despite the recognized role of jumping ability performance, there is limited research examining how preseason training affects vertical jump metrics in professional basketball players. The available evidence is largely based on non-elite athletes, limiting their generalizability. Understanding how preseason conditioning influences neuromuscular performance in professionals is crucial for optimizing training protocols and enhancing on-court performance [7].

Therefore, the aim of this study was to analyze changes in sprinting and jumping performance in professional basketball players during the preseason and to determine to what extent these neuromuscular adaptations are influenced by playing position (Guards vs. Bigs). We hypothesized that sprinting and jumping performance would improve over the course of the preseason, with position-specific differences arising from the distinct physiological and functional demands associated with each role. Understanding these adaptations may assist strength and conditioning coaches in designing more targeted training programs to enhance player readiness for the competitive season.

## 2. Materials and Methods

### 2.1. Study Design

A quasi-experimental study design was carried out to examine the effect of a 6-week training program on the neuromuscular and athletic performance of professional basketball players. Given the applied nature of the study within a professional setting, no control group was included. All participants underwent the same training intervention, which was tailored to their individual characteristics and needs to optimize adaptations and performance gains.

### 2.2. Participants

One hundred and six professional male basketball players from European leagues (German Basketball Bundesliga, Basketball Champions League, and Spanish Basketball League) participated in this study. Based on their playing positions, 59 players were classified as Guards (point guards, shooting guards, and small forwards), and 47 as Bigs (power forwards and centers) (Table 1). All players were injury-free and provided informed consent prior to the testing. The study was approved by the local institutional ethics committee and conducted in accordance with the Declaration of Helsinki (64th WMA General Assembly, Fortaleza, Brazil, 2013).

### 2.3. Procedure

Basketball players underwent two sprinting and jumping assessments during preseason separated by 6 weeks. Both sprinting and jumping assessments were carried out on the first day of a microcycle, after a rest day, and at least 48 h after the most recent match. Testing took place on an indoor basketball court under controlled environmental conditions that conformed to standards set by the International Basketball Federation (FIBA) [28], including a temperature range of 16–22 °C and relative humidity between 40 and 60%. Data collection occurred in August within a climate-controlled facility to avoid excessive heat and ensure consistency across sessions.

#### 2.3.1. Sprinting Test

Prior to performing the sprint tests, all players completed a standardized warm-up protocol consisting of two progressive sprints of 10 and 5 m at approximately 80% and 90% of their maximal sprint velocity, respectively. Sprint performance was assessed with a local position measurement tracking system by two pairs of dual-beam photocells (Witty Wireless Trainer Timer^®^, Microgate Srl, Bolzano, Italy), a system previously shown itself to be valid and reliable for monitoring sprint distance and time [29]. Each player performed two attempts per sprinting test, ensuring full recovery between trials. The fastest time was retained for subsequent statistical analysis.

##### Linear Sprint (5, 10 and 20 m)

Linear sprints were conducted over distances of 5, 10, and 20 m. Players began from a stationary position just before the timing gates, ensuring they did not prematurely activate the sensors. The start was self-initiated, with no external signal, and randomized to prevent anticipation. To maintain maximal effort through the finish line, two cones were placed one meter beyond the final gate, providing a visual cue to discourage deceleration prior to completion.

##### Change of Direction (COD)

The 505 Agility Test was employed to assess COD ability by measuring acceleration deceleration and turning speed [30]. From a stationary start, players sprinted forward through a 10 m timing gate (Witty Wireless Trainer Timer^®^ system, Microgate Srl, Bolzano, Italy), continued to the 15 m line, executed a rapid 180° turn, and sprinted back through the same gate. Each player performed the test twice, turning off once with each foot (left and right). The recorded time, from the initial gate pass to the return pass, was used as a performance indicator of agility [31].

##### Curved Sprint

**Figure 1:**
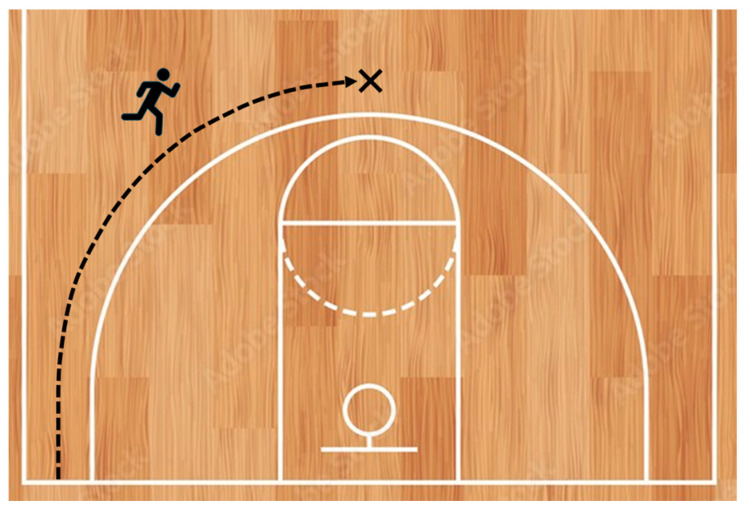
Runing trajectory of the curved sprint test.

In this novel assessment of sprinting ability, players began in the corner of the basketball court, just beyond the baseline, and performed a maximal-effort sprint directed toward the mid-point of the three-point arc (see Figure 1). The test was conducted bilaterally, with players starting from both left and right corners. To standardize movement execution, participants were instructed not to step on the three-point line at any point during the sprint.

#### 2.3.2. Jumping Tests

Prior to the jump testing, all players performed two preparatory attempts for each jump type: two submaximal trials (at approximately 80% and 90% of perceived effort) for the SJ, CMJ, and CMJA, and one submaximal trial for the SHDJ and the LBJ. No warm-up trial was performed for the 15-s Repeated Jump Test.

Vertical jump performance was measured via flight time using a Chronojump BoscoSystem contact platform (v.1.7.0 for Windows, CHRONOJUMP Boscosystem^®^, Barcelona, Spain), which has demonstrated excellent reliability for vertical jump assessment (ICC = 0.95) [32]. For horizontal jumps, a retractable 3 m tape measure (Stanley^®^ PowerLock, Stanley Black & Decker Inc., New Britain, CT, USA) was used [33]. Two trials were conducted for each test, with the best attempt retained for analysis.

##### Squat Jump (SJ)

The SJ was performed following the protocol described by De Blas et al. [32]. Players began in a self-selected semi-squat position, which they held for two seconds before jumping on a verbal command. While knee angle was not standardized, players naturally adopted positions between 90 and 100°, a range previously shown to not significantly affect performance [10]. Hands were placed on the hips throughout the movement to eliminate arm contribution and minimize elastic energy storage, thereby isolating concentric lower-limb power.

##### Countermovement Jump (CMJ)

The CMJ followed the same protocol as the SJ but without the isometric pause. Players performed a rapid downward movement followed by a maximal vertical jump. Hands remained on the hips to prevent upper-body involvement, allowing for assessment of both concentric strength and stretch-shortening cycle efficiency [9,34].

##### Arm-Swing Countermovement Jump (CMJA)

The CMJA allowed full use of the upper limbs. Participants performed a self-paced countermovement and were instructed to jump as high as possible. This variation assessed concentric and elastic power, as well as coordination and movement efficiency under sport-specific conditions [9].

##### Single-Hop for Distance Jump (SHDJ)

For the SHDJ, players started from a single-leg stance on the take-off leg and jumped as far forward as possible, landing on both feet. The test followed the protocol described by Schmitt et al. [35]. This landing strategy minimized knee loading and reduced potential inhibitory effects from fear of unilateral landing.

##### Lateral Bound Jump (LBJ)

The LBJ was performed by jumping one-legged laterally to the contralateral side, landing on a single foot in a stable and controlled manner [15]. Two trials were completed on each side, and the best result was retained.

##### Single-Leg Repeated Jumps (SLRJ)

The SLRJ was adapted from Wen et al.’s [36] protocol. Players performed repeated jumps from a one-legged position for 15 s, aiming to maximize jump height while minimizing ground contact time. The test was repeated on the opposite leg following full recovery. This test allowed not only assessing jump height, but also reactive strength index (RSI) and fatigue accumulation over time.

#### 2.3.3. Preseason Training Program

The 6-week preseason training program was designed to enhance basketball players’ performance through an integrated approach that combined technical–tactical training with strength and neuromuscular development. Training predominantly involved high-frequency basketball-specific drills, with a strong emphasis on improving tactical execution and technical skills. Strength and conditioning sessions were systematically incorporated throughout the preseason to target key physical capacities, including maximal strength, upper- and lower-body power, and upper-body hypertrophy (see Table 2).

The core of the preseason program focused on basketball-specific technical and tactical drills, conducted six to eight times per week under the supervision of the head and assistant coaches. These sessions aimed to enhance decision-making, team coordination, and overall game readiness by simulating competitive scenarios and situational play.

Concurrently, strength and power training were performed two to three times per week and were structured around three key components of physical development (see Table 3):

- Maximal strength (primarily lower body): Including exercises such as squats, single-leg squats, deadlift, hip thrust.
- Explosive power and rate of force development (RFD): Implemented through loaded jumps, Olympic lifts, medicine ball throws, and exercises using accommodated resistance.
- Upper-body strength and hypertrophy: Involving movements such as bench press, pull-ups, rows, and shoulder press.

Although the preseason training program followed a common framework, with all players completing the same number of on-court group sessions and strength-training sessions in the weight room, the specific training loads and content were individualized. Individualization was primarily based on three criteria: (1) baseline performance profile identified during the initial assessment (e.g., maximal strength, power, mobility); (2) history of previous injuries, to prevent overload and re-injury; and (3) daily monitoring of acute discomfort or fatigue, which guided load adjustments on a session-by-session basis. This approach ensured that all athletes adhered to a standardized training structure while simultaneously receiving tailored adjustments aimed at optimizing performance and minimizing injury risk.

**Figure 2:**
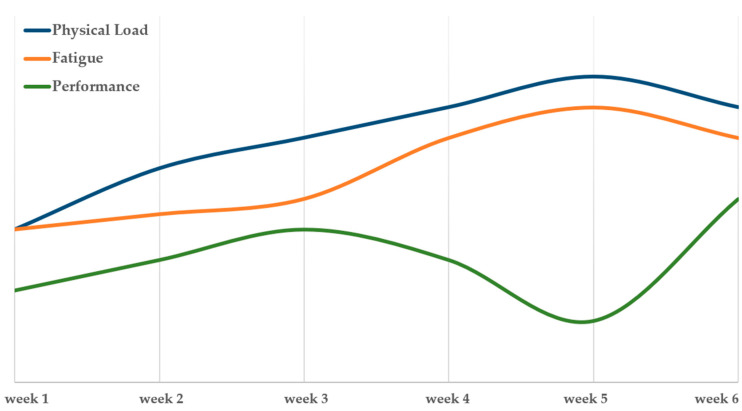
Basketball players’ estimated training load, fatigue, and athletic performance during preseason from week 1 to 6.

Training load was monitored using a subjective 0–10 rating scale, applied weekly to estimate perceived physical exertion. In parallel, perceived athletic performance and cumulative fatigue were also assessed using the same scale throughout the 6-week preseason period. This approach provided a practical and continuous overview of players’ training responses and adaptive trends over time (see Figure 2).

All physical training sessions were supervised by certified strength and conditioning coaches to ensure proper technique, individualized progression, and optimal load management. Emphasis was consistently placed on executing all movements with maximally intended velocity to maximize neuromuscular adaptations. However, the specific training content and exercise selection varied depending on the targeted physical quality of each session (see Table 3), allowing for a periodized approach aligned with performance goals.

### 2.4. Statistical Analysis

Relative reliability was assessed using the intraclass correlation coefficient (ICC), calculated via a two-way mixed-effects model (single measures, absolute agreement). ICC values were interpreted as follows: <0.5 poor reliability, 0.5–0.75 moderate reliability, 0.75–0.9 good reliability, and >0.9 excellent reliability [37]. The coefficient of variation (CV) was also computed as a measure of absolute reliability [38].

To verify assumptions for parametric testing, normality was assessed using the Shapiro–Wilk test, with statistical significance set at *p* < 0.05. All variables met the criteria for normality and homoscedasticity.

To evaluate the study hypotheses, a mixed-design factorial analysis of variance (mixed ANOVA) was conducted to examine performance changes across time and playing position. The within-subject factor was Time (pre- vs. post-preseason assessment), and the between-subject factor was Group (Guards vs. Bigs). Effect sizes were reported using partial eta squared (η^2^_p_) and interpreted as small (≥0.01), moderate (≥0.06), or large (≥0.14). Effect sizes were reported using partial eta squared (η^2^_p_) and interpreted as small (≥0.01), moderate (≥0.06), or large (≥0.14). All statistical analyses were performed using SPSS version 26.0 (IBM Corp., Armonk, NY, USA), with the level of significance set at *p* < 0.05.

## 3. Results

Reliability analyses indicated good to excellent reproducibility for most sprint and jump tests (see Table 4). Linear sprint test showed ICC values of 0.81 (0.70–0.89) and a CV of 0.1% for 5 m; 0.91 (0.86–95) and a CV of 0.1% for 10 m; and 0.92 (0.87–0.95) and a CV of 0.1% for 20 m, confirming strong measurement stability. Curved sprint displayed slightly lower ICCs, but still within acceptable limits, with ICC of 0.79 (0.67–0.86) for the right side and 0.84 (0.75–0.90) left side, as well as a CV of 0.1% for both sides.

For jumping tests, ICC values were generally high, showing ICC values of 0.96 (0.77–0.98) and a CV of 1.5% for SJ; 0.93 (0.84–0.96) and a CV of 2.6% for CMJ; and 0.97 (0.70–0.99) and a CV of 1.4% for CMJA. For SHDJ, an ICC of 0.76 (0.52–0.88) right leg and 0.80 (0.58–0.90) left leg and a CV of 2.8% and 2.2%, respectively, were obtained. For LBJ, an ICC of 0.87 (0.72–0.93) right leg and 0.78 (0.55–0.89) left leg and a CV of 1.5% and 2.3%, respectively, were observed. For SLRJ, an ICC of 0.83 (0.55–0.96) right leg and 0.84 (0.59–0.94) left leg and a CV of 1.7% and 1.9%, respectively, were recorded. The wider ICC value for SHDJ and SLRJ suggest lower robustness and warrants cautious interpretation, although CVs remained acceptable (1.7–2.8%).

As shown in Table 4, players’ linear sprint performance demonstrated clear improvements after preseason training. The largest gain occurred in the 5 m sprint with a left-leg start, showing a time reduction of 3.53% (*p* = 0.001; η^2^_p_ = 0.370). Meaningful improvements were also observed in the 10 m sprint with both right- and left-leg starts (−2.37%; *p* = 0.001; η^2^_p_ = 0.239 and −2.17%; *p* = 0.001; η^2^_p_ = 0.362, respectively), while the 20 m sprint showed smaller but still relevant gains (−0.88%; *p* = 0.001; η^2^_p_ = 0.278), all reflecting large effect sizes.

Curved sprint performance improved in both directions, with a moderate effect size to the right (−2.35%; η^2^_p_ = 0.159) and a large effect size to the left (−4.52%; η^2^_p_ = 0.418). In contrast, COD performance showed moderate effect sizes (η^2^_p_= 0.122–0.662), yet the changes were not statistically significant, suggesting potential adaptations of practical but inconclusive magnitude.

In jumping performance, the most consistent improvements were observed in vertical jumps involving the stretch-shortening cycle. The CMJ moderately improved by 3.53% (*p* = 0.05; η^2^_p_ = 0.079), while the CMJA showed a more substantial increase of 4.84% with a moderate-to-large effect (η^2^_p_ = 0.166). The LBJ with the right leg improved significantly (1.68%; η^2^_p_ = 0.188), reflecting a large effect size, whereas SHDJ and SLRJ displayed only slight, non-significant gains, with small-to-moderate effects. No relevant changes were observed in the SJ.

When considering playing position (see Table 5), no significant interaction effects were found between playing position (Bigs vs. Guards) for linear sprint, COD, or curved sprint. Bigs were slower than Guards in the 20 m sprint both at the beginning (3.42%; *p* = 0.036; η^2^_p_ = 0.113), with a moderate effect size. Additionally, Guards outperformed Bigs in vertical jumping, showing consistently higher CMJ values at both baseline (+5.66%) and post-preseason (+6.48%), with moderate effect sizes (η^2^_p_ = 0.079). Guards also improved more in CMJA compared with Bigs (6.85% vs. 1.87%; η^2^_p_ = 0.123), representing the only significant *time* × *position interaction*.

No significant interaction effects were observed between time and playing position for linear sprinting, Change of Direction (COD), or curved sprint performance. The only significant interaction was found in the CMJA, where Guards demonstrated greater improvements than Bigs over the preseason period (6.85% vs. 1.87%; *p* = 0.02; η^2^_p_ = 0.123), indicating a moderate effect size.

## 4. Discussion

The main findings of this study indicate that after a 6-week preseason training program, professional basketball players improved their linear and curved sprint performance, as well as their jumping ability in the CMJ, CMJA, and right-leg LBJ. In contrast, no significant improvements were observed in COD, SJ, SHDJ or SLRJ.

Significant improvements were observed in the 5 m sprint from a left-leg start (3.53%), and in the 10 m sprint for both right (2.37%, *p* = 0.001) and left-leg start (2.17%, *p* = 0.001), with the largest improvement in the 5 m left-leg sprint. Performance also improved in the 20 m sprint (0.88%, *p* = 0.001), though with a smaller magnitude. In the curved sprint, players improved in both directions (right: 2.35%, *p* = 0.024; left: 4.52%, *p* = 0.001). However, COD performance did not change significantly despite moderate effect sizes (η^2^_p_ = 0.122 and 0.662, right and left turn, respectively).

These sprint-related improvements are consistent with previous studies suggesting that the early preseason training in professional basketball may enhance neuromuscular qualities related to acceleration and short sprinting ability [39,40]. In particular, the largest improvements in this study were found during the acceleration phase (5 to 10 m). These improvements likely reflect adaptations in concentric force production and technical refinements promoted by the training process [41].

While 20 m sprint performance also improved, the smaller gain suggests limited adaptation in maximal velocity. This aligns with findings that maximal velocity development typically requires longer sprint distances (≥30 m) and more targeted sprint training for speed development [42]. Additionally, Lesinski et al. [43] similarly reported that while strength training leads to moderate improvements in strength and jump, its impact on linear sprinting, particularly top speed, tends to be modest, suggesting a lower training sensitivity of maximal velocity compared to other neuromuscular qualities more related with rate of force development such as acceleration or jump performance.

Curved sprint performance showed meaningful improvements, especially on the left side, which is relevant given that curved sprinting accounts for roughly one-third of all basketball sprint actions [44]. This may indicate enhanced lateral force generation, coordination, and motor control, qualities that may not be adequately stimulated by linear sprint drills alone [45]. Yet, COD performance did not follow the same pattern, possibly due to insufficient specificity of training stimuli or a ceiling effect, as elite players often demonstrate high baseline COD proficiency [46,47]. Research suggests that targeted eccentric overload and high-intensity decelerations are required to meaningfully improve COD ability [48], which may have been underrepresented in this program. The absence of significant improvements in COD, despite moderate effect sizes, may reflect the limited specificity of preseason training for deceleration and re-acceleration mechanics. In addition, COD testing in elite athletes may lack the sensitivity to detect small but practically meaningful changes. Similar findings have been reported in recent studies, where plyometric interventions improved strength, sprint, and jump performance but not COD, likely due to insufficient training volume or specificity [49,50].

Regarding jump performance, significant gains were found in CMJ (3.53%, *p* = 0.05) and CMJA (4.84%, *p* = 0.001), with the latter showing the greatest improvement. No significant differences were found in SJ, SHDJ or LBJ, except for the right-leg LBJ (1.68%, *p* = 0.027). While small-to-moderate effect sizes were noted for SLRJ, these changes were not significant. The greater improvements in CMJ and CMJA suggest that preseason training predominantly enhanced stretch-shortening cycle efficiency rather than isolated concentric strength [51]. The superior gain in CMJA compared to CMJ may reflect benefits of arm swing, including improved upper-body coordination and core stability, contributing to whole-body momentum generation [52].

The absence of significant SJ improvements suggests that the training stimulus was more aligned with elastic energy utilization and neuromuscular coordination rather than maximal concentric force development. This interpretation aligns with previous studies reporting that CMJ is generally more sensitive than SJ to plyometric and ballistic training interventions [53]. Conversely, it contrasts with findings from strength-oriented programs, where greater improvements in SJ compared to CMJ have been reported [54]. Therefore, the lack of meaningful SJ gains in our cohort may reflect that the preseason training did not sufficiently emphasize maximal concentric force production at low contraction velocities.

Similarly, the absence of significant improvements in SHDJ and SLRJ further indicates limited enhancement of horizontal and unilateral force production, despite the use of single-leg exercises in the training program. This outcome may be explained by an insufficient training volume or a lack of specificity for unilateral and horizontal tasks, which are essential for efficient propulsion and landing control [19]. However, the isolated improvement in the right-leg LBJ could reflect asymmetrical neuromuscular adaptations, a common phenomenon in team sport athletes due to habitual side dominance and sport-specific movement [55].

Interestingly, although unilateral exercises were included in the preseason program, improvements were more consistent in bilateral vertical jumps (CMJ and CMJA) than in unilateral jumps. This finding suggests that the training volume and intensity may not have been sufficient to induce meaningful unilateral adaptations, which is consistent with previous evidence indicating that greater targeted loads are required to drive adaptations in unilateral and horizontal capacities [46].

From a positional standpoint, no significant differences were found between Bigs and Guards in linear sprinting, COD, or curved sprinting performance. However, Guards exhibited greater CMJ performance at baseline (5.66%) and post-preseason training program (6.48%), with a moderate effect size. Furthermore, Guards showed significantly greater gains in CMJA performance compared with Bigs (6.85% vs. 1.87%, *p* = 0.02). These results may reflect positional differences in upper-body coordination and explosive power, with Guards typically showing superior relative jumping ability [19]. Overall, both positions benefited from preseason training, but Guards may be more responsive in improving vertical jump performance, particularly in jump variations involving the stretch-shortening cycle and upper-limb contribution.

These results are broadly in line with previous studies reporting preseason improvements in CMJ and CMJA without large performance differences across playing positions [7]. However, evidence remains mixed on whether game-related fatigue reduces or preserves jump performance. On one hand, it has been suggested that vertical jump performance is relatively stable, even under acute fatigue conditions during competition or training [27], while other authors have shown that simulated games can indeed reduce jump performance [56].

The influence of training load and season structure on jump performance has also been explored. Evidence suggests that vertical jump metrics are generally maintained across the competitive season, with only minor fluctuations related to fatigue and training load [11]. These authors also emphasized the importance of monitoring variables beyond jump height alone to obtain a more comprehensive picture of neuromuscular status. On the other hand, interventions studies have demonstrated improvements in functional performance and reductions in inter-limb asymmetries, with the magnitude of adaptation depending on the specific direction of force targeted [57]. Resistance training combined with plyometric methods has shown considerable efficacy in enhancing vertical jump performance, especially in younger or less experienced athletes [58]. For instance, Barrera-Domínguez et al. [59] reported substantial gains in jumping ability following an 8-week strength program based on force–velocity profiling, although the participants were not professional players. It is worth noting that neuromuscular performance improvements have become increasingly difficult in elite populations due to their already high training status [60,61].

Finally, the short-term nature of this study raises questions about how these gains translate to in-season performance. This issue is particularly relevant in team sports like basketball, where athletes are simultaneously exposed to endurance- and strength-oriented stimuli. Although concurrent training can enhance overall physical fitness, it may also trigger an interference effect, most evident in hypertrophic adaptations, while neuromuscular outcomes such as strength and power appear less affected when training variables are carefully managed [62,63]. During short preseason periods, where athletes are expected to achieve rapid improvements across multiple domains, managing this potential interference becomes especially critical.

The recent literature has highlighted strategies to mitigate these potential conflicts associated with concurrent training. Proper sequencing of training modalities, adequate nutritional support (especially carbohydrate and protein intake), strategic scheduling (e.g., prioritizing strength work earlier in the day), and individualized rest and recovery protocols have all been shown to optimize dual adaptations in strength and endurance [64,65]. Such programming considerations are especially relevant in professional basketball, where athletes must develop and maintain anaerobic power, aerobic capacity, and neuromuscular readiness. Future studies should therefore examine how concurrent training during preseason affects not only performance metrics but also fatigue management, recovery, and injury risk throughout the competitive season.

Several limitations of this study should be acknowledged. First, the absence of a control group limits the ability to establish causal relationships between preseason training and the observed performance changes. While such designs are challenging in applied professional settings, this limitation reduces the strength of inference and warrants a cautious interpretation of the findings. Second, the relatively short intervention period (6 weeks) may not fully capture longer-term adaptations or the sustainability of performance gains during the competitive season. Third, despite the relatively large sample of professional athletes from different European leagues, the findings may not generalize to other competitive levels or to female players. Fourth, given the high training status of participants, ceiling effects may have attenuated the magnitude of adaptation, particularly in tasks such as COD and unilateral jumping. Finally, the ICC confidence intervals for some tests (e.g., SLRJ) were wide, suggesting lower robustness and a need for cautious interpretation of these specific measurements. Future studies employing stronger comparative designs (e.g., randomized or within-subject protocols) and longer observation periods are needed to strengthen causal inference and clarify how preseason training translates into in-season performance outcomes.

## 5. Practical Applications

From a practical perspective, strength and conditioning coaches should consider implementing position-specific strategies that reflect the distinct physical demands of Guards and Bigs. Given the limited duration of preseason and the expectation of rapid performance gains, players should arrive in adequate baseline physical condition to maximize adaptations. Future research should explore the long-term effects of preseason programs on in-game performance, injury prevention, and recovery dynamics. Furthermore, individualized training prescriptions based on playing position, training history, and physical profile may represent an effective strategy to optimize development and reduce injury risk.

## 6. Conclusions

The results of this study suggest that a 6-week preseason training program integrating technical–tactical practice with strength and neuromuscular training in professional basketball players may lead to meaningful improvements in linear and curved sprinting performance, as well as in jumping ability, particularly in tasks involving the stretch-shortening cycle such as the CMJ and CMJA. In contrast, no clear gains were observed in COD performance or unilateral horizontal jumps, indicating that these qualities may require more specific training stimulus. Positional differences appear minimal, although Guards may be more responsive than Bigs in improving vertical jump performance, particularly when arm swing is involved.

These findings should be interpreted with caution given the absence of a control group and the exploratory nature of the design. Nonetheless, they provide practical insights into how preseason training may contribute to neuromuscular readiness in elite basketball players and highlight the importance of tailoring training strategies to both performance domains and positional demands. Future studies employing stronger comparative designs and longer follow-up periods are needed to clarify the causal impact of preseason training and its transfer to in-season performance.

## Figures and Tables

**Table 1:** Descriptive statistics of basketball players’ body characteristics.

	Guards		Bigs	
	Assessment 1	Assessment 2	Assessment 1	Assessment 2
Age (years)	26.04 ± 2.16	26.15 ± 2.16	26.75 ± 2.7	26.87 ± 2.7
Body Mass (kg)	84.03 ± 8.13	84.39 ± 8.2	107.34 ± 7.25	104.94 ± 4.87
Height (cm)	189 ± 7.6	189 ± 7.6	207 ± 5.6	207 ± 5.6
Fat (%)	5.91 ± 3.9	4.53 ± 2.9	10.40 ± 4.3	8.28 ± 3.9
Body Mass Index	23.52 ± 1.72	23.62 ± 1.46	25.05 ± 1.56	24.49 ± 1.48
Abdominal Skinfold (mm)	8.32 ± 5.5	7.21 ± 4.39	14.88 ± 6.19	12.38 ± 6.51
Quadriceps Skinfold (mm)	5.61 ± 3.62	6.54 ± 3.22	11.21 ± 6.03	9.31 ± 4.78

Data is reported as mean value ± standard deviation. Skinfolds were obtained following the measurement standards of the ISAK. Fat percentage (%) was obtained using Jackson & Pollock [27] formula from three skinfolds.

**Table 2:** Average weekly training volume based on training content.

Training Time	Load (Hours)
Technical–tactical team sessions	14 h
Lifting room	2 h
Individual technique	2 h
Individual physical training	1 h
Games	2 h
Total	20 h

**Table 3:** Preseason training program content.

Training Focus	Sets	Reps	Intensity	Primary Exercises
Lower body maximal strength	2	4–6	RIR 1–3	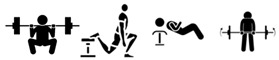
Explosive strength and power	2	6–8	RIR 6–8	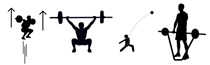
Upper body hypertrophy	3	8–10	RIR 0–2	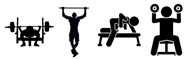

Reps (repetition), RIR (repetitions in reserve).

**Table 4:** Sprinting and jumping performance differences between assessments points.

Variable				Assessment 1	Assessment 2	Diff (%)	ES
Sprinting Test	Linear Sprint	5 m	Right	1.16 ± 0.08 (1.13–1.18)	1.14 ± 0.07 (1.12–1.16)	−1.12	0.005
			Left	1.16 ± 0.08 (1.14–1.18)	1.12 ± 0.07 (1.09–1.13)	−3.53 *	0.370
		10 m	Right	1.90 ± 0.096 (1.88–1.93)	1.85 ± 0.08 (1.83–1.87)	−2.37 *	0.239
			Left	1.89 ± 0.09 (1.87–1.92)	1.85 ± 0.08 (1.82–1.87)	−2.17 *	0.362
		20 m		3.15 ± 0.13 (3.12–3.18)	3.12 ± 0.12 (3.08–3.15)	−0.88 *	0.278
	Curved Sprint		Right	2.28 ± 0.10 (2.23–2.29)	2.22 ± 0.10 (2.18–2.25)	−2.46 *	0.159
			Left	2.30 ± 0.10 (2.26–2.32)	2.20 ± 0.09 (2.16–2.22)	−4.53 *	0.418
	COD		Right	2.29 ± 0.10 (2.25–2.32)	2.22 ± 0.11 (2.17–2.26)	−3.08	0.122
			Left	2.34 ± 0.13 (2.26–2.38)	2.24 ± 0.08 (2.20–2.27)	−4.36	0.066
Jumping Test	SJ			39.15 ± 4.76 (38.0–40.7)	40.64 ± 5.09 (39.1–42.1)	3.55	0.040
	CMJ			42.49 ± 4.78 (41.3–44.0)	43.99 ± 5.38 (42.4–45.5)	3.53 *	0.079
	CMJA			50.78 ± 5.79 (49.0–52.4)	53.23 ± 6.87 (51.2–55.2)	4.84 *	0.166
	SHDJ		Right	229.19 ± 17.80 (222.7–235.6)	233.03 ± 19.45 (226.0–240.0)	1.68	0.024
			Left	235.28 ± 19.39 (228.2–242.3)	240.41 ± 18.11 (233.8–246.9)	2.18	0.047
	LBJ		Right	213.83 ± 15.07 (208.2–219.4)	217.33 ± 15.87 (211.4–223.2)	1.64 *	0.188
			Left	218.80 ± 16.01 (212.7–224.8)	219.27 ± 16.13 (213.2–225.2)	0.21	0.008
	SLRJ		Right	15.16 ± 3.56 (13.4–16.6)	16.54 ± 3.95 (14.9–18.8)	9.08	0.026
			Left	15.39 ± 3.14 (14.1–16.8)	16.60 ± 2.99 (15.2–17.9)	7.86	0.088

Data is reported as mean ± standard deviation (95% Confidence Interval). ES (Effect Size), COD (Change of Direction), SJ (Squat Jump), CMJ (Countermovement Jump), CMJA (Arm-Swing Countermovement), SHDJ (Single-Leg Hop for Distance Test), LBJ (Lateral Bound Jump), SLRJ (Single-Leg Repeated Jumps). * *p* < 0.05, significantly different between pre and post.

**Table 5:** Sprinting and jumping performance differences between assessments based on playing positions.

Variable				Bigs			Guards			ES
				Assessment 1	Assessment 2	Diff (%)	Assessment 1	Assessment 2	Diff (%)	
Sprinting Test	Linear Sprint	5 m	Right	1.18 ± 0.07 (1.15–1.22)	1.17 ± 0.06 (1.14–1.19)	−1.32	1.13 ± 0.07 (1.10–1.15)	1.12 ± 0.07 (1.09–1.14)	−0.95	0.002
			Left	1.19 ± 0.08 (1.17–1.24)	1.15 ± 0.06 (1.12–1.17)	−3.35	1.14 ± 0.07 (1.11–1.15)	1.09 ± 0.06 (1.06–1.11)	−3.70	0.043
		10 m	Right	1.94 ± 0.09 (1.90–1.98)	1.89 ± 0.07 (1.86–1.92)	−2.30	1.87 ± 0.09 (1.84–1.191)	1.83 ± 0.09 (1.79–1.85)	−2.43	0.038
			Left	1.93 ± 0.09 (1.90–1.98)	1.89 ± 0.08 (1.85–1.91)	−2.42	1.86 ± 0.08 (1.83–1.89)	1.82 ± 0.07 (1.78–1.84)	−1.93	0.012
		20 m		3.21 ± 0.13 (3.14–3.25)	3.19 ± 0.09 (3.14–3.24)	−0.62	3.10 ± 0.11 (3.07–3.15)	3.07 ± 0.11 (3.02–3.10)	−1.15	0.113
	Curved Sprint		Right	2.30 ± 0.10 (2.24–2.33)	2.25 ± 0.10 (2.20–2.29)	−2.26	2.25 ± 0.10 (2.19–2.28)	2.19 ± 0.10 (2.13–2.23)	−2.46	0.023
			Left	2.33 ± 0.10 (2.28–2.37)	2.23 ± 0.07 (2.29–2.26)	−4.49	2.27 ± 0.09 (2.21–2.30)	2.16 ± 0.10 (2.10–2.20)	−4.57	0.042
	COD		Right	2.29 ± 0.09 (2.23–2.34)	2.24 ± 0.10 (2.18–2.29)	−2.15	2.28 ± 0.10 (2.22–2.34)	2.18 ± 0.13 (2.11–2.29)	−4.33	0.097
			Left	2.35 ± 0.10 (2.28–2.41)	2.26 ± 0.05 (2.22–2.29)	−3.65	2.32 ± 0.16 (2.19–2.40)	2.20 ± 0.09 (2.15–2.27)	−5.31	0.026
Jumping Test	SJ			38.30 ± 4.85 (36.1–40.4)	39.55 ± 4.77 (37.4–41.6)	3.28	39.93 ± 4.65 (38.3–42.1)	41.45 ± 5.30 (39.4–43.7)	3.81	0.058
	CMJ			41.13 ± 4.42 (39.3–42.9)	42.37 ± 4.69 (40.3–44.4)	3.02	43.60 ± 4.84 (41.9–45.8)	45.31 ± 5.62 (43.1–47.4)	3.92	0.079
	CMJA			50.23 ± 6.38 (47.3–53.0)	51.19 ± 7.41 (47.8–54.4)	1.91	51.26 ± 5.31 (49.0–53.4)	55.04 ± 5.93 (52.5–57.4)	7.36 *	0.044
	SHDJ		Right	228.50 ± 12.15 (221.4–235.5)	235.21 ± 10.62 (229.0–241.3)	2.93	229.72 ± 21.54 (219.0–240.4)	231.33 ± 24.41 (219.1–243.4)	0.70	0.052
			Left	233.29 ± 15.30 (224.4–242.1)	239.64 ± 8.85 (234.5–244.7)	2.72	236.83 ± 22.37 (225.7–247.9)	241.00 ± 23.18 (229.4–252.5)	1.76	0.021
	LBJ		Right	213.21 ± 9.79 (207.5–218.8)	221.79 ± 11.14 (215.3–228.2)	4.02	214.38 ± 18.84 (204.3–224.4)	213.44 ± 18.56 (203.5–223.3)	−0.44	0.012
			Left	216.79 ± 12.07 (209.8–223.7)	222.43 ± 13.59 (214.5–230.2)	2.60	220.56 ± 19.02 (210.4–230.7)	216.50 ± 18.03 (206.8–226.1)	−1.84	0.019
	SLRJ		Right	14.60 ± 3.39 (12.1–18.1)	15.56 ± 3.70 (12.9–18.2)	6.58	15.78 ± 3.83 (12.8–18.1)	17.62 ± 4.14 (15.1–21.3)	11.66	0.063
			Left	14.37 ± 3.46 (11.8–16.8)	15.19 ± 2.31 (13.5–16.8)	5.70	16.40 ± 2.58 (14.8–17.9)	18.01 ± 3.01 (15.8–20.1)	9.82	0.191

Data is reported as mean ± standard deviation (95% Confidence Interval). ES (Effect Size), COD (Change of Direction), SJ (Squat Jump), CMJ (Countermovement Jump), CMJA (Arm-Swing Countermovement), SHDJ (Single-Leg Hop for Distance Test), LBJ (Lateral Bound Jump), SLRJ (Single-Leg Repeated Jumps). * *p* < 0.05, significantly different between pre and post.

## Data Availability

The data that support the findings of this study are available from the corresponding author, upon reasonable request.

## Abbreviations

The following abbreviations are used in this manuscript:

CMJ	Countermovement Jump
CMJA	Arm-Swing Countermovement Jump
COD	Change of Direction
LBJ	Lateral Bound Jump
SHDJ	Single-Hop for Distance Jump
SJ	Squat Jump
SLRJ	Single-Leg Repeated Jumps
